# A Retrospective Study on Neonatal Jaundice: Early Risk Stratification Value of DAT‐FAT Serological Profiles Confirmed by AET

**DOI:** 10.1002/kjm2.70253

**Published:** 2026-06-17

**Authors:** Tian‐Ge Wu, Yan‐Feng Zhang, Zhong‐Jun Shen, Yao Li, Ning Xu, Xiao‐Yi Liu, Li‐Yan Zhao, Ming‐Hao Shi

**Affiliations:** ^1^ Clinical Transfusion Department The Second Hospital of Jilin University Changchun Jilin China

**Keywords:** hemolytic disease of the newborn, neonatal jaundice, risk stratification, serological markers, serological panel

## Abstract

This retrospective study aimed to explore the value of DAT‐FAT serological profiles confirmed by AET in classifying neonatal jaundice, evaluating its severity, and guiding clinical management. A total of 915 jaundiced newborns (584 pathological, 331 physiological) admitted from July 2018 to August 2021 were included. Univariate and multivariate logistic regression analyses were conducted to identify risk factors. To assess predictive ability for jaundice type and early risk stratification of jaundice severity, the 426 patients with ABO incompatibility were stratified into subgroups based on the results of the DAT‐FAT‐AET serological panel. Maternal pregnancy count, gestational weeks, direct bilirubin at admission, and ABO incompatibility were identified as independent predictors of jaundice type (*p* < 0.05). Those who tested triple‐positive in the DAT‐FAT‐AET serological panel (Group 2) had the highest predictive value for pathological jaundice (area under the curve, 0.920 [0.788–0.994]), indicating the maximum severity of pathological jaundice (*p* < 0.01). AET serves as the definitive criterion for confirming ABO‐incompatible jaundice, while DAT‐FAT profiles enable early risk stratification of severity. Subgrouping by AET‐confirmed DAT‐FAT serology helps assess etiology and severity in ABO‐incompatible neonatal jaundice, supporting clinical early risk stratification.

**Abbreviations**
ABO‐HDN: ABO hemolytic disease of the newborn
AET: antibody elution test
CI: confidence Interval
DAT: direct antiglobulin Test
DBIL: direct bilirubin
FAT: free antibody test
HDN: hemolytic disease of the newborn
IVIG: intravenous immunoglobulin
OR: odds ratio
ROC: receiver operating characteristic
SD: standard deviation
TBIL: comparison of serum total bilirubin
TSB: total serum bilirubin

## 1 Introduction

Neonatal jaundice, which is defined as elevated blood bilirubin levels, is a common clinical symptom among newborns that can be categorized into physiological and pathological types [1, 2]. In most neonates, jaundice appears on the second or third day after birth and is accompanied by varying degrees of anemia [3]. Around 60%–85% of newborns usually develop jaundice, which is mostly physiological and requires no intervention. However, pathological jaundice (with unique causes and clinical importance) must not be overlooked considering its potential to advance if left untreated [4, 5]. Pathological jaundice among newborns has been attributed to numerous causes, including bacterial infections, biliary atresia, perinatal asphyxia, and prematurity [6, 7, 8]. Another common factor for pathological jaundice is hemolytic disease of the newborn (HDN) [9, 10, 11]. HDN mainly comes from maternal–infant blood type incompatibility: maternal antifetal erythrocyte IgG crosses the placenta, triggering immune reactions that destroy fetal/neonatal erythrocytes. Studies have shown that ABO incompatibility is a major cause of neonatal pathological jaundice. Hence, early accurate prediction, diagnosis, and intervention for ABO‐HDN‐induced pathological jaundice are crucial for good neonatal outcomes.

Current laboratory serological testing for ABO incompatibility‐related HDN mainly includes the direct antiglobulin test (DAT), free antibody test (FAT), and antibody elution test (AET). Regarding their clinical applications, all three tests can be performed in most laboratories equipped with basic immunoserological testing capabilities. Although the equipment and reagents required for DAT and FAT are quite common, professional laboratory personnel are needed for accurate interpretation of results. In contrast, AET is more complex and requires well‐equipped laboratories [12, 13]. Given that most studies have focused solely on the sensitivity of the DAT in diagnosing ABO incompatibility, limited research is available on the correlation between the DAT‐FAT‐AET serological panel and neonatal pathological jaundice [14]. As such, the current study aimed to investigate the clinical value of the serological panel in the early risk stratification of the causes, types, and severity of neonatal jaundice.

## 2 Materials and Methods

### 2.1 General Information

All implemented procedures were compliant with the ethical norms set by relevant human experimentation committees (both institutional and national), as well as with the Helsinki Declaration of 1964 and its subsequent revisions. After performing a database search, a total of 1468 patients with neonatal jaundice treated at the author's hospital between July 2018 and August 2021 were identified.

### 2.2 Diagnostic Criteria for Jaundice Types

Pathological jaundice in newborns refers to an increase in bilirubin load or a decrease in bilirubin clearance, resulting in a total serum bilirubin (TSB) level higher than 220.5 μmol/L, or meeting one of the following conditions: appearance of jaundice within 24 h after birth; a daily increase in TSB level of > 85.5 μmol/L or an hourly increase in TSB level of > 8.5 μmol/L; and a conjugated bilirubin level > 25.6–34 μmol/L.

Physiological jaundice in newborns is a transient condition caused by the characteristics of bilirubin metabolism. It usually appears on the 2nd to 3rd day after birth, reaches its peak on the 4th to 6th day, and resolves within 2 weeks in term infants and within 3 to 4 weeks in preterm infants, with the TSB level not exceeding 220.5 μmol/L.

### 2.3 Inclusion Criteria for the Study Group

The study group included [1] those who met the diagnostic criteria for pathological jaundice and [2] those who had completed laboratory tests related to HDN, including maternal, neonatal, DAT, FAT, and AET. Infants with incomplete clinical data or those suffering from two or more diseases simultaneously were excluded from the study group.

### 2.4 Inclusion Criteria for the Control Group

The control group included those who exhibited clinical signs of physiological jaundice. Those who had hemolysis due to ABO/Rh incompatibility, as well as congenital diseases and other organ abnormalities were excluded from the control group.

### 2.5 Reagent and Instrument

ABO blood grouping cards (microcolumn gel assay), ABO standard erythrocytes, and BX‐1 type elution solutions were provided by Changchun Bioxun Biotechnology Co. Ltd. The antihuman globulin assay card (microcolumn gel assay) was provided by Diagnostic S.A. The full‐automated blood type analyzer was provided by Hamilton.

### 2.6 Identification of Blood Groups

The microcolumn gel method was applied for ABO blood typing of mothers and infants. Agglutination of the tested red blood cells with monoclonal IgM anti‐A/anti‐B reagents in the gel, with agglutinated cells remaining on the gel surface or interstices after centrifugation, indicated a positive result (indicating the presence of corresponding antigens), whereas the sinking of the cells to the gel column bottom indicated a negative result [8].

### 2.7 DAT‐FAT‐AET Serological Panel

Microcolumn gel‐based DAT‐FAT‐AET serological panel was performed for EDTA‐K_2_ anticoagulated whole blood specimens with maternal–fetal ABO incompatibility. Given that this is a retrospective analysis, the timing of sample collection was defined as follows: all tests were performed upon the admission of neonates aged 0–14 days (after jaundice onset), based on their clinical records. The testing procedures are described as follows:

### 2.8 DAT

This test involved adding 50 μL of 0.8%–1% neonatal RBC suspension to the IgG microcolumn gel card, after which centrifugation was performed at 900 r/min for 2 min and then at 1500 r/min for 3 min to detect RBC‐sensitizing blood group antibodies.

### 2.9 FAT

This test involved adding 25 μL neonatal plasma and 50 μL of 0.8%–1% standard RBCs to A, B, and O‐labeled wells of microcolumn gel antihuman globulin card. Thereafter, samples were incubated at 37°C and centrifuged at 900 r/min for 2 min and then at 1500 r/min for 3 min to detect incompatible blood group antibodies in serum.

### 2.10 AET

This test involved adding wash test RBCs four times. Acid elution solution was prepared by mixing elution solutions A and B at a ratio of 4:1. Thereafter, 250 μL of test RBCs were mixed with the solution, incubated at room temperature for 1–2 min, and centrifuged at 3000 rpm for 1 min. The supernatant was then neutralized and centrifuged again to obtain the eluate. Thereafter, 50 μL of 0.5%–0.8% known‐type RBCs and 50 μL eluate were added to antihuman globulin card wells, incubated at 37°C for 15 min, and centrifuged at 900 rpm for 2 min and at 1500 rpm for 3 min. The results were visually read to detect RBC‐bound sensitizing antibodies.

According to the criterion set for the upper limit of physiological jaundice (i.e., TSB > 220.5 μmol/L) from Chapter 13 of Practice of Pediatrics (8th Edition) [15], basic data, relevant test indicators, and the results of the serological panel for ABO incompatibility were compared and analyzed for different types of jaundice among the included newborns.

### 2.11 Assessment of Jaundice Severity

According to the TSB level, neonatal jaundice can be defined as mild (100–200 μmol/L), moderate (201–300 μmol/L), and severe (> 300 μmol/L).

### 2.12 Treatment Methods for Jaundice

The treatment strategies for ABO incompatibility‐related neonatal jaundice mainly encompass phototherapy (blue light therapy), medication (intravenous immunoglobulin), and exchange transfusion. This study focused on determining the correlation between serological test results and phototherapy. According to Chapter 13 of Practice of Pediatrics (8th Edition) [15], blue light therapy required a blue light source between 425 and 475 nm delivered through a phototherapy unit that maintains a standard light intensity of 8–10 μW/(cm^2^ nm).

### 2.13 Clinical Outcome Evaluation

Given the focus of our study on subgroup analysis according to length of hospitalization, the clinical outcomes of neonates were evaluated using the length of hospitalization as the primary objective indicator.

### 2.14 Prognosis Evaluation

Prognosis was assessed based on whether jaundiced neonates met discharge criteria after blue light therapy. Accordingly, a good prognosis is characterized based on the following factors: return of serum bilirubin levels to the normal range for the corresponding age without recurrence; normal heart rate and transcutaneous oxygen saturation during electrocardiogram and oxygen monitoring; self‐feeding ability, normal urination, defecation, and bowel sounds; significant reduction in skin jaundice; and a reasonable length of hospitalization.

### 2.15 Statistical Analysis

Statistical analyses were conducted using SPSS 27.0 and R 4.3.1. Continuous variables were tested for normality of distribution (Shapiro–Wilk test) and homogeneity of variance (Levene's test). Normally distributed and variance‐homogeneous continuous variables were presented as mean ± standard deviation compared using independent‐samples *t*‐test, whereas non‐normally distributed or variance‐heterogeneous continuous variables were expressed as medians (interquartile ranges) and compared using the Mann–Whitney U test. Categorical variables are presented as numbers (percentages) and compared using the chi‐square test or Fisher's exact test.

Univariable and multivariable logistic regression analyses were performed to identify independent risk factors for neonatal pathological jaundice, and a nomogram prediction model was constructed. Model discrimination was evaluated using receiver operating characteristic (ROC) curve analysis with calculation of sensitivity, specificity, positive/negative predictive values. Calibration was assessed via calibration curves.

Prolonged hospitalization was defined as > 5 days (based on the study population's median length of stay), with associated factors being analyzed using univariable/multivariable logistic regression. Multiple‐group comparisons were performed using one‐way analysis of variance, whereas ordered categorical variables were analyzed via ordinal logistic regression. All tests were two‐sided, with *p* < 0.05 indicating statistical significance.

## 3 Results

### 3.1 Clinical Data Analysis of 915 Patients With Neonatal Jaundice

A total of 1468 patients with neonatal jaundice from the author's hospital were included in this study. Among them, 553 patients who did not meet the inclusion criteria were excluded: 100 due to incomplete clinical data, 298 due to congenital diseases or significant organ dysfunction, and 155 with incomplete test results. Ultimately, 915 patients were enrolled in this retrospective study and divided into the study group (584 patients with pathological jaundice) and the control group (331 patients with physiological jaundice). The case screening process is shown in Figure S1.

As shown in Table S1, significant differences in some variables, including hospitalization duration of newborns, birth weight, maternal pregnancy count, gestational weeks, hospitalization days, TSB at admission, direct bilirubin at admission, hemoglobin at admission, admission diagnosis, maternal blood type, neonatal blood type, and whether blood transfusion was performed, were found between the pathological and physiological jaundice groups (*p* < 0.05). These differences are crucial for the early identification of neonatal jaundice types and provide theoretical support for the scientific grouping of subsequent serological tests.

### 3.2 Independent Predictors of Pathological Jaundice

Our results showed that factors, such as hospitalization duration, birth weight, maternal pregnancy count, gestational weeks, hospitalization days, direct bilirubin at admission, hemoglobin at admission, admission diagnosis, maternal blood type‐O, neonatal blood type AB, and whether blood transfusion was required, were significantly associated with the type of jaundice (*p* < 0.05). Moreover, both univariate and multivariate logistic regression analyses indicated that maternal pregnancy count, gestational weeks, direct bilirubin at admission, and ABO incompatibility were independent predictors of the type of jaundice (*p* < 0.05; Table 1). These findings lay the foundation for a comprehensive exploration of the pathogenesis of neonatal jaundice and the construction of a risk assessment model.

**TABLE 1:** Univariate and multivariate logistic regression for neonates with jaundice.

Variable	Univariable analysis			Multivariable analysis		
	Odds ratio	95% CI	*p*	Odds ratio	95% CI	*p*
Hospitalization duration of newborns (days)	1.139	1.074–1.207	< 0.001	1.041	0.988–1.121	0.134
Birth weight (g)	1.001	1.000–1.001	< 0.001	1.000	0.989–1.011	0.911
Maternal pregnancy count (time)	1.175	1.029–1.342	0.017	1.218	1.066–1.329	0.011
Gestational weeks	1.140	1.087–1.196	< 0.001	1.114	1.009–1.231	0.032
Hospitalization days	1.112	1.058–1.905	0.034	1.105	0.876–1.519	0.217
Direct bilirubin at admission (μmol/L)	1.315	1.252–1.381	< 0.001	1.253	1.198–1.396	< 0.001
Hemoglobin at admission (g/L)	0.993	0.986–1.000	0.048	1.011	0.997–1.016	0.153
Leukocyte count at admission (×10^9^/L)	1.002	0.994–1.011	0.629			
Gender (female)	0.847	0.647–1.110	0.230			
Mode of delivery (vaginal delivery)	1.122	0.833–1.511	0.448			
Admission diagnosis (ABO incompatible)	2.982	2.239–3.971	< 0.001	3.211	1.418–7.125	0.008
Maternal blood type (B)	1.029	0.700–1.513	0.883	3.175	0.799–10.135	0.110
Maternal blood type (AB)	0.497	0.190–1.300	0.154	0.877	0.289–2.916	0.852
Maternal blood type (O)	1.533	1.147–2.050	0.004	1.036	0.440–2.411	0.930
Neonatal blood type						
Neonatal blood type (B)	1.153	0.868–1.532	0.325	0.511	0.326–1.861	0.551
Neonatal blood type (AB)	0.841	0.482–1.468	0.543	1.452	0.691–2.923	0.330
Neonatal blood type (O)	0.411	0.295–0.572	< 0.001	0.837	0.403–2.118	0.851
Blood transfusion (yes)	6.548	1.993–21.519	0.002	4.008	0.943–14.932	0.119

*Note:* Other diagnoses refer to premature infants, expiratory dyspnea, and neonatal infection.

Abbreviation: CI, confidence interval.

### 3.3 Construction and Evaluation of the Nomogram Model

**FIGURE 1:**
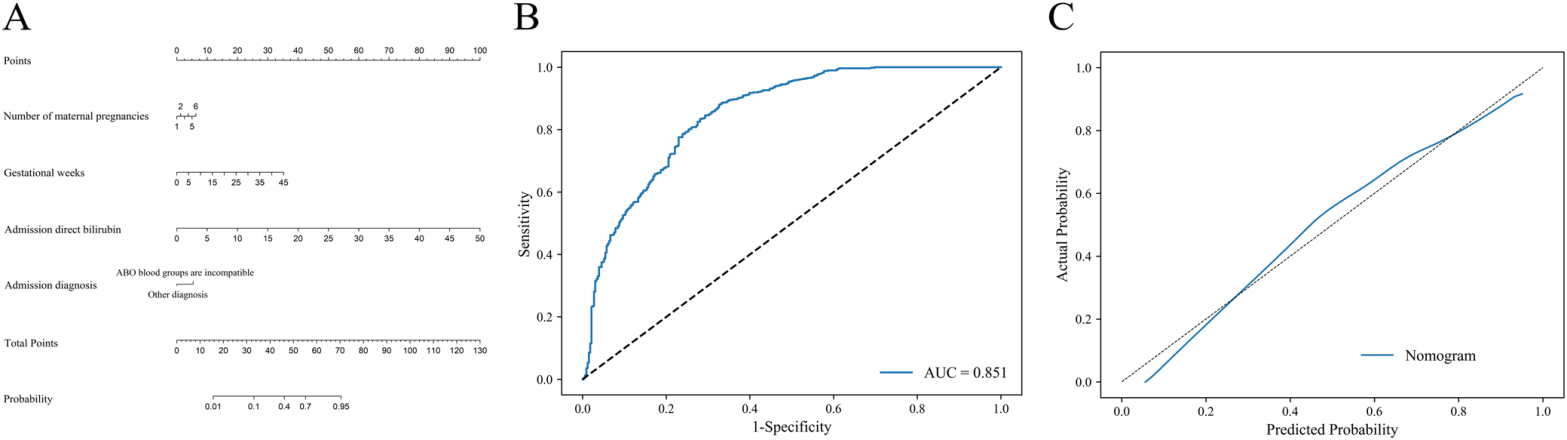
Construction and evaluation of the nomogram model. (A) Nomogram for predicting pathological jaundice incorporating the following independent predictors: Maternal pregnancy count, gestational weeks, admission direct bilirubin, and ABO incompatibility. (B) Receiver operating characteristic curve (ROC) for pathological jaundice predicted by the nomogram showing an area under the curve (AUC) of 0.851 (95% CI: 0.825, 0.876). (C) Calibration curve demonstrating the agreement between the predicted and observed probabilities of pathological jaundice (Hosmer–Lemeshow test, *p* = 0.215).

A nomogram model involving independent predictors was constructed using multivariate logistic regression, which well predicted the type of jaundice with an area under the curve (AUC) of 0.851 (95% CI: 0.825, 0.876) (Figure 1A,B). Moreover, calibration curve analysis demonstrated that the nomogram model exhibited a satisfying calibration performance (Hosmer–Lemeshow test: *p* = 0.215) (Figure 1C).

### 3.4 Role of the Serological Panel in Neonatal Jaundice With ABO Incompatibility

Serological testing, including DAT, FAT, and AET, was immediately performed on 426 neonates with ABO incompatibility‐related jaundice upon admission. Based on these results, the neonates were divided into Group 1 (negative FAT and positive DAT and AET), Group 2 (positive DAT, FAT, and AET), Group 3 (negative DAT and positive FAT and AET), and Group 4 (negative DAT and FAT and positive AET). A positive AET result is a definitive diagnostic criterion for confirming that neonatal jaundice is caused by ABO incompatibility rather than other etiologies. Therefore, patients with negative AET results were excluded from the analysis, as they could not be confirmed to have ABO incompatibility‐induced jaundice.

### 3.5 Predictive Value of the Serological Panel for Pathological Jaundice in Newborns With ABO Incompatibility

**FIGURE 2:**
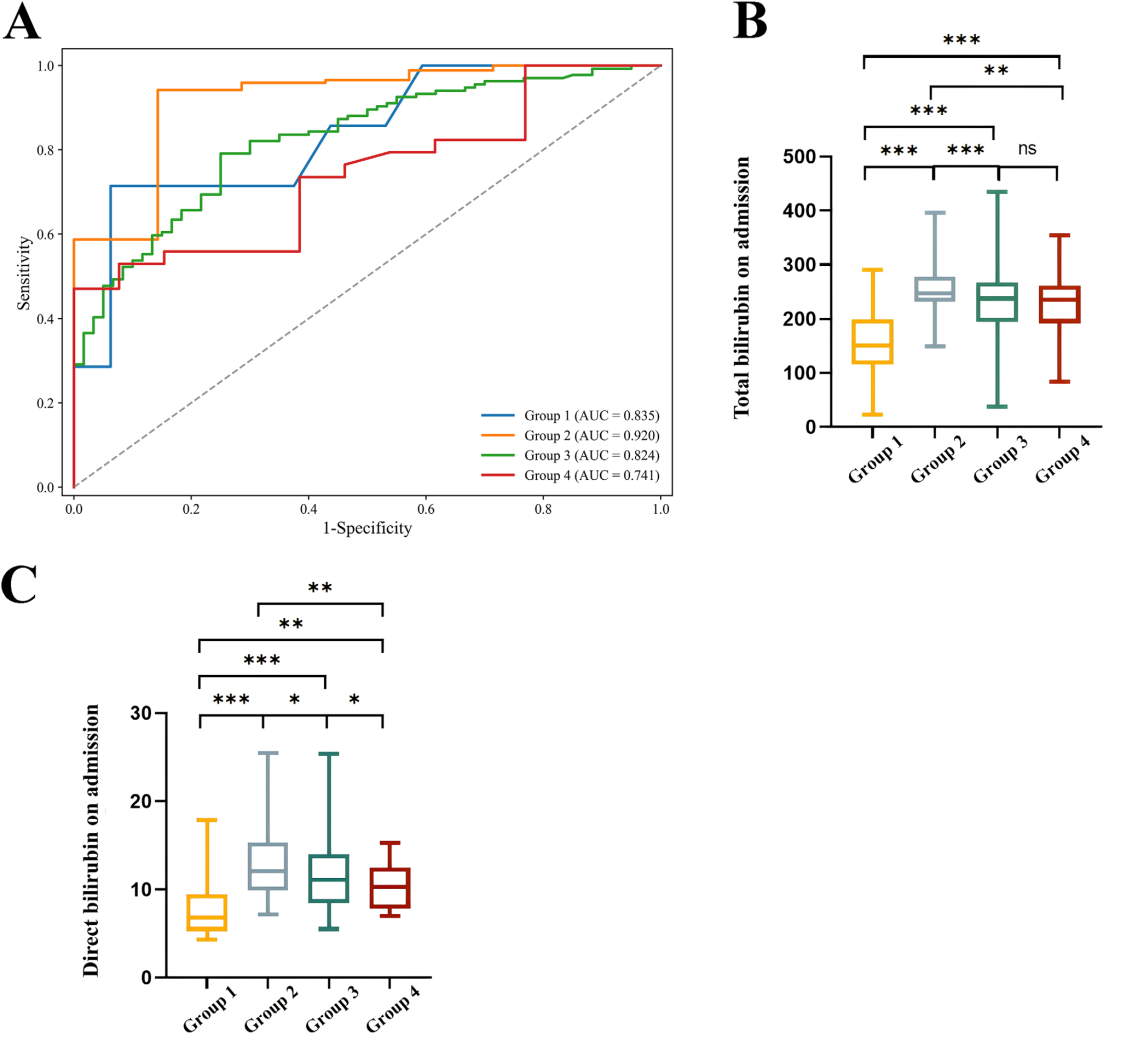
Analysis of indicators related to neonatal pathological jaundice in different subgroups. (A) Receiver operating characteristic curve (ROC) curve analysis of serological subgroups for predicting pathological jaundice in neonates with ABO incompatibility. (B and C) Total bilirubin (TBIL) and direct bilirubin (DBIL) levels at admission across subgroups defined based on serological test results.

Statistical analysis showed that the AUC of Group 2 was 0.920 (95% CI: 0.788–0.994), with a sensitivity, specificity, and accuracy of 0.942, 0.857, and 0.939, respectively (Table 2, Figure 2A). This finding indicates that triple positivity in the serological panel had an extremely high predictive value for diagnosing pathological jaundice in neonates with ABO incompatibility.

**TABLE 2:** ROC analysis of DAT‐FAT‐AET combined serological testing for pathological jaundice in newborns with ABO incompatibility.

Model	AUC (95% CI)	Sensitivity	Specificity	Accuracy	PPV	NPV
Group1	0.835 (0.638–0.994)	0.714 (0.333–1.000)	0.938 (0.844–0.983)	0.897 (0.795–0.974)	0.714 (0.533–0.961)	0.938 (0.848–0.972)
Group2	0.920 (0.788–0.994)	0.942 (0.906–0.976)	0.857 (0.597–0.961)	0.939 (0.905–0.972)	0.994 (0.981–0.996)	0.375 (0.142–0.611)
Group3	0.824 (0.765–0.882)	0.791 (0.715–0.860)	0.750 (0.640–0.857)	0.778 (0.716–0.835)	0.876 (0.812–0.935)	0.616 (0.494–0.726)
Group4	0.741 (0.590–0.874)	0.471 (0.305–0.641)	1.000 (1.000–1.000)	0.617 (0.489–0.745)	1.000 (1.000–1.000)	0.419 (0.242–0.586)

*Note:* Group 1: Direct Antiglobulin Test positive, Free Antibody Test negative, Antibody Elution Test positive; Group 2: Direct Antiglobulin Test positive, Free Antibody Test positive, Antibody Elution Test positive; Group 3: Direct Antiglobulin Test negative, Free Antibody Test positive, Antibody Elution Test positive; Group 4: Direct Antiglobulin Test negative, Free Antibody Test negative, Antibody Elution Test positive.

Abbreviations: AUC, area under the curve; CI, confidence interval; NPV, negative predictive value; PPV, positive predictive value; ROC, receiver operating characteristic.

Moreover, univariate analysis revealed significant differences in TSB and direct serum bilirubin (DSB) levels at admission among different subgroups of ABO‐incompatible neonates (*p* < 0.05). Accordingly, TSB and DSB levels at admission were significantly lower in Group 1 than in other subgroups, whereas both bilirubin levels were significantly higher in Group 2 than in other subgroups. These results confirm that subgroup differences in serological testing exert important effects on the severity of neonatal jaundice (Figure 2B,C).

Subsequent statistical analysis of the association between different subgroups and the severity of pathological jaundice demonstrated significant differences in the distribution of jaundice severity (mild, moderate, and severe) among subgroups (*χ* ^2^ = 101.8, *p* < 0.01). Specifically, the probability of severe pathological jaundice was significantly low in Group 1 and markedly high in Group 2 relative to the rest of the groups (Table S2), further confirming that the three serological tests allow reliable prediction of jaundice type and early risk stratification for assessing disease severity.

### 3.6 Value of the Serological Panel in Predicting Clinical Outcomes of Neonatal Jaundice With ABO Incompatibility

Statistical analysis of serological testing, admission TSB, DSB levels, and phototherapy efficacy in subgroups of jaundiced newborns with ABO incompatibility was performed. The results of the chi‐square test indicated a significant difference in the effectiveness of phototherapy among subgroups (*χ* ^2^ = 7.936, *p* < 0.05). This finding suggests that the DAT‐FAT‐AET serological panel has a specific predictive value for the effectiveness of phototherapy in treating neonatal jaundice in subgroups. Notably, Group 2 (i.e., those who tested positive for all three serological tests) showed significantly higher mean TSB and DSB levels than did the other groups while having the highest phototherapy rate, confirming more severe jaundice manifestations. This finding indicates that the DAT‐FAT‐AET serological panel has good consistency in predicting disease severity.

Subsequent univariate analysis showed that subgroups with positive serological test results (Groups 2, 3, and 4) had a significantly increased risk of prolonged hospitalization compared to Group 1 (control group) (all *p* < 0.01). However, in the multivariate model, TSB at admission (OR: 1.005, *p* = 0.028), phototherapy (OR: 2.197, *p* = 0.017), and gestational age (OR: 0.828, *p* = 0.024) emerged as independent predictors of prolonged hospitalization, whereas the prognostic value of each serological subgroup showed marginal significance (*p* values ranging from 0.078 to 0.130) (Table S3). The findings of our statistical analyses were fully consistent with the disease severity of each subgroup, which suggests that those with a more severe condition had a higher risk for prolonged hospitalization. This finding indicates that the serological panel has good performance in risk stratification for disease severity.

## 4 Discussion

The current study aimed to explore whether the serological panel could serve as an intuitive, convenient, and effective screening method for the early determination of the etiology and classification of jaundice types, as well as for risk stratification of disease severity among neonates with pathological jaundice.

Notably, the retrospective design of the current study suggests that no participant trials or interventions were conducted. Instead, a nomogram model was developed to predict the risk of neonatal pathological jaundice. The model's predictive performance was evaluated using the ROC curve, which yielded an AUC of 0.851 (95% CI: 0.825–0.876), indicating high predictive efficiency and underscoring the significance of the included predictors in jaundice classification. Meanwhile, the model demonstrated that ABO incompatibility was an independent risk factor for neonatal pathological jaundice, consistent with the findings from the global literature [16, 17, 18, 19].

The current study followed the 2022 AAP updated guideline for neonatal hyperbilirubinemia, which employs gestational age‐ and hour‐specific bilirubin nomograms and raises phototherapy thresholds to reduce overtreatment [20]. Although our single TSB cutoff of > 220.5 μmol/L does not fully align with current stratification standards, the core finding that the DAT‐FAT‐AET serological panel was positively correlated with elevated bilirubin and increased disease severity remains robust and clinically relevant.

Currently, diagnosing neonatal jaundice type and severity mainly relies on continuous observation and multiple testing of bilirubin or transcutaneous bilirubin levels among in‐hospital patients. A review of the literature shows that international studies often use positive DAT for early prediction of elevated serum bilirubin levels [21, 22, 23]. Some propose using DAT to screen for ABO‐incompatibility‐induced jaundice and treat positive DAT newborns [21, 24], whereas others argue that conducting DAT on all newborns is a waste of resources [25].

Therefore, we sought to determine the utility of the serological panel through statistical analysis. The positivity rate of DAT for detecting incomplete antibodies on red blood cells was only 50%, which may be attributed to the underdevelopment of ABO blood antigens on neonatal red blood cell membranes. In contrast, the positivity rate of FAT for detecting free antibodies in serum was 81.26%, with a positive result indicating a potential risk for hemolysis in vivo. However, positive FAT results only indicate the presence of antibodies in infants without necessarily confirming sensitization and thus cannot serve as the sole basis for diagnosis. AET positivity is a key criterion for confirming neonatal ABO hemolytic disease (ABO‐HDN) and the primary diagnostic basis for excluding neonatal jaundice caused by other etiologies. Overall, the serological test panel improves the accuracy of detecting pathological jaundice induced by ABO incompatibility.

The serological panel revealed that Group 2 (all positive) had an extremely high positive predictive value for ABO incompatibility‐related neonatal pathological jaundice. The three serological tests showed that Group 2 had an AUC of 0.920 (95% CI: 0.788–0.994), with a sensitivity, specificity, and accuracy of 0.942, 0.857, and 0.939, respectively. Notably, Group 2 exhibited a high positive and negative predictive value of 0.994 and 0.375, respectively. Therefore, this subgroup demonstrated the highest predictive value for ABO incompatibility‐related neonatal pathological jaundice. The positivity rate of pathological jaundice in Group 2 was 98.28%, which was significantly higher than that of other subgroups. Ordered logistic regression analysis of the serological panel and clinical indicators, such as direct bilirubin at admission and length of hospitalization, showed that only TSB and DSB levels at admission exhibited significant differences. Moreover, in children who tested positive for all three tests (Group 2), the TSB and DSB at admission were significantly higher than those in other groups (*p* < 0.0001). In summary, our results indicate that neonates who test positive in all three tests have pathological jaundice and have significantly increased probability of having moderate to severe jaundice, especially severe jaundice. Notably, although Group 2 showed a relatively high predictive value for pathological jaundice, other positive groups, including Groups 3 and 4, should also be considered in clinical screening to reduce missed diagnoses. Future studies should explore more sensitive combined indicator systems to enhance predictive efficiency. Additionally, serological test results act as critical screening tools for diagnostic classification and prognostic assessment. Univariate logistic regression analysis showed that positive serological subgroups (Groups 2–4) had a significantly higher risk of prolonged hospitalization than did the reference Group 1 (all *p* < 0.01). This finding further validates the clinical value of positive serological test results in predicting severe jaundice and prolonged hospitalization. Treatment decisions primarily rely on absolute bilirubin levels, whereas the serological panel assists in evaluating the etiology and disease severity rather than dictating phototherapy initiation. However, the chi‐square test revealed significant differences in phototherapy effectiveness across subgroups (*p* < 0.05), indicating that serological classification of different subgroups could predict the efficacy of phototherapy in neonatal jaundice, which is partially consistent with international literature reporting that DAT‐positive neonates had increased bilirubin levels, early jaundice onset, and an increased need for phototherapy [26]. Conversely, other reports argued that some newborns with negative DAT results also require phototherapy, suggesting that DAT alone is insufficient for determining the presence of pathological jaundice or guiding clinical treatment decisions [27]. Our analysis also confirmed that the serological panel demonstrated good performance in risk stratification for predicting the severity of neonatal jaundice. However, it should be noted that serological results may indirectly influence prognosis by verifying bilirubin levels and treatment intensity, rather than serving as an independent prognostic factor.

More positive serological results correlated with increased erythrocyte antibody binding, which indicates predisposition to moderate to severe pathological jaundice requiring timely intervention. The current study reveals that the serological panel, which can be completed within 40 min, reflects greater antibody involvement via higher positive rates, which increases severe jaundice risk.

However, this retrospective study has several limitations. First, the retrospective design precluded adjustment for confounders, showing only associations without causality. Second, temporal heterogeneity in testing duration may have affected positivity rates and subgroup assignment. Third, the small number of samples collected from a single center limits the generalizability of our findings, and AET was restricted to acid elution. Future prospective studies that include standardized timing, larger multicenter cohorts, and diverse AET methods are needed to explore the dynamic correlations between serological indicators and clinical parameters. These efforts will provide supporting evidence for early risk assessment of jaundice, thereby optimizing early risk stratification for the severity of neonatal jaundice.

## 5 Conclusion

The DAT‐FAT‐AET serological panel can be used to effectively determine the etiologies and types of jaundice among neonates with maternal–fetal ABO blood group incompatibility. This method can accurately identify the association between jaundice and neonatal hemolytic diseases, simplify the classification system, and enable rapid and reliable early risk stratification for the severity of neonatal disease. Based on these findings, clinicians can promptly clarify the etiology, type, and severity of jaundice, providing an effective basis for early risk assessment of neonatal jaundice.

## Funding

This work was supported by Science and Technology Development Plan of Jilin Province, 20230204103YY and 20240401068YY.

## Ethics Statement

Approved by The Ethics Committee of The Second Hospital of Jilin University (2021058).

## Conflicts of Interest

The authors declare no conflicts of interest.

## Supporting information


**Figure S1:** Flowchart showing the selection of patients with neonatal jaundice. Schematic diagram of the inclusion and exclusion process for patients with neonatal jaundice included in this study. Newborns were screened based on clinical symptoms, gestational age, serum bilirubin levels, and completeness of serological test results. A total of 1468 cases were initially identified, with 553 being excluded due to incomplete data, congenital diseases, or missing test results. Ultimately, 915 eligible patients (584 pathological jaundice, 331 physiological jaundice) were included for analysis.

**Table S1:** Baseline Characteristics of 915 Neonates with Jaundice.

**Table S2:** Logistic Regression Model Analysis of Jaundice Severity in Subgroups.

**Table S3:** Univariate and Multivariate Logistic Regression Analyses of Prolonged Hospitalization in Neonates with Jaundice.

## Data Availability

The data that support the findings of this study are available from the corresponding author upon reasonable request.
