# Supplementary figures and images for: A Retrospective Study on Neonatal Jaundice: Early Risk Stratification Value of DAT‐FAT Serological Profiles Confirmed by AET
Source: Kaohsiung J Med Sci. 2026 Jun 17:e70253. Online ahead of print. doi: 10.1002/kjm2.70253 (PMC13399740; doi:10.1002/kjm2.70253)

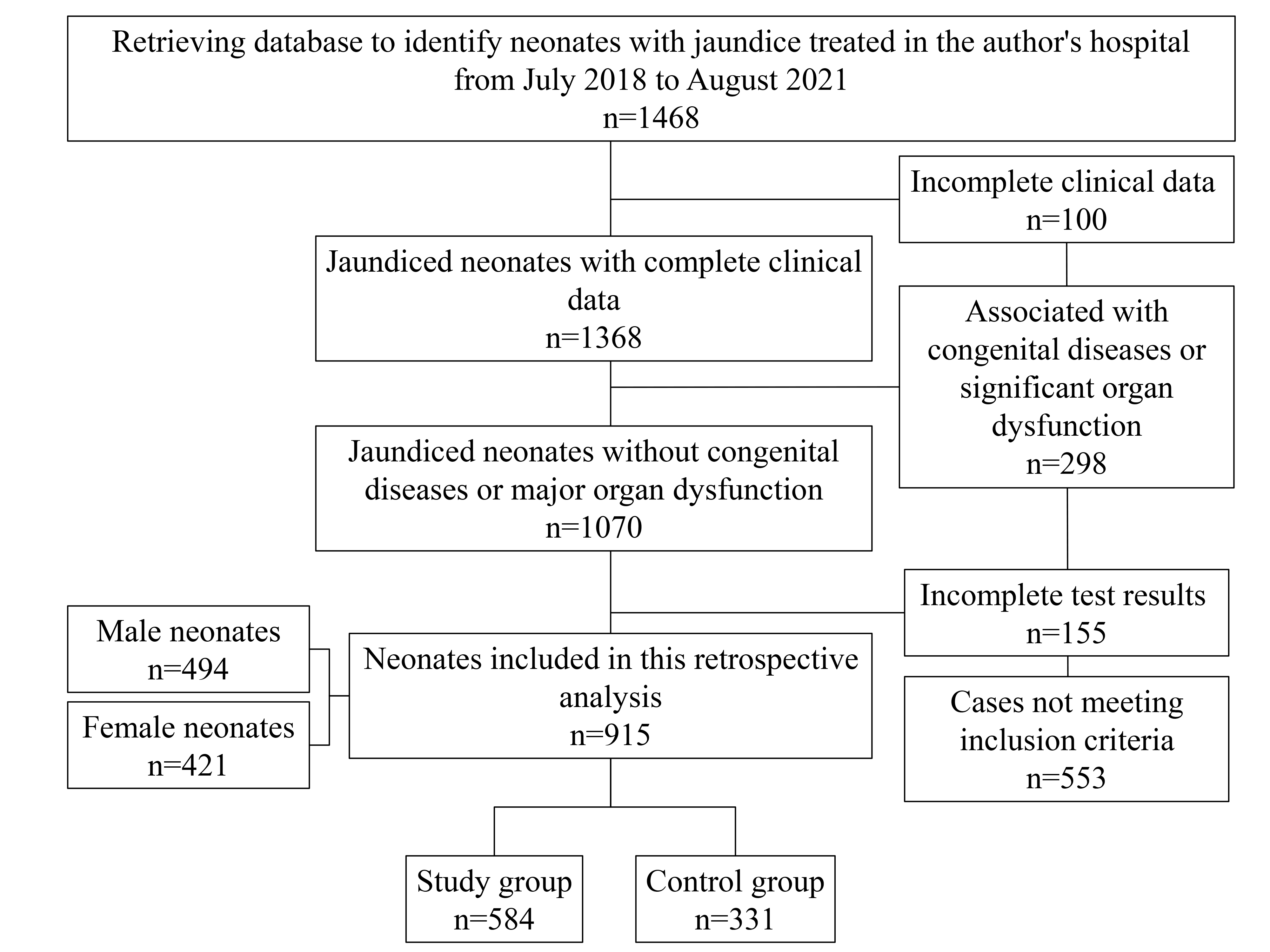

Supplement: Supplementary file 1 — Figure S1: Flowchart showing the selection of patients with neonatal jaundice. Schematic diagram of the inclusion and exclusion process for patients with neonatal jaundice included in this study. Newborns were screened based on clinical symptoms, gestational age, serum bilirubin levels, and completeness of serological test results. A total of 1468 cases were initially identified, with 553 being excluded due to incomplete data, congenital diseases, or missing test results. Ultimately, 915 eligible patients (584 pathological jaundice, 331 physiological jaundice) were included for analysis. [file KJM2-9999-e70253-s001.jpg]
