# Supplementary material for: A Retrospective Study on Neonatal Jaundice: Early Risk Stratification Value of DAT‐FAT Serological Profiles Confirmed by AET
Source: Kaohsiung J Med Sci. 2026 Jun 17:e70253. Online ahead of print. doi: 10.1002/kjm2.70253 (PMC13399740; doi:10.1002/kjm2.70253)
Supplement: Supplementary file 2 — Table S1: Baseline Characteristics of 915 Neonates with Jaundice. [file KJM2-9999-e70253-s003.docx]

**Table S1 Baseline Characteristics of 915 Neonates with Jaundice**

| Variable | Pathological Jaundice  (*n* = 584) | Physiological Jaundice  (*n* = 331) | *P* value |
| --- | --- | --- | --- |
| Age | 2.00 (1.00-4.00) | 3.00 (1.00-4.00) | 0.441 |
| Hospitalization duration of newborns (d) | 2.00 (0.00-4.00) | 1.00 (0-2.00) | < 0.001 |
| Birth weight (g) | 3150.00 (2700.00-3530.00) | 2950.00 (2200-3400.00) | < 0.001 |
| Maternal pregnancy count (time) | 2.00 (1.00-2.00) | 2.00 (1-2.00) | < 0.001 |
| Gestational weeks | 37.00 (36.00-39.00) | 37.00 (34-38.00) | < 0.001 |
| Hospitalization days | 8.00 (6.00-14.00) | 5.00 (4.00-11.00) | 0.046 |
| Total bilirubin at admission (μmol/L) | 259.95 (238.31-276.31) | 146.80 (102-189.92) | < 0.001 |
| Direct bilirubin at admission (μmol/L) | 11.88 (9.80-14.66) | 8.46 (6-10.52) | < 0.001 |
| Hemoglobin at admission (g/L) | 169.00 (153.00-181.00) | 172.00 (160-181.00) | < 0.001 |
| Leukocyte count at admission ( × 10^9^/L) | 9.70 (8.30-11.30) | 10.20 (9-11.20) | 0.133 |
| Gender |  |  | 0.257 |
| Male | 324 (55.5) | 170 (51.4) |  |
| Female | 260 (44.5) | 161 (48.6) |  |
| Mode of delivery |  |  | 0.494 |
| Cesarean section | 406 (69.5) | 238 (71.9) |  |
| Vaginal delivery | 178 (30.5) | 93 (28.1) |  |
| Admission diagnosis |  |  | < 0.001 |
| Other diagnoses | 257 (44.0) | 232 (70.1) |  |
| ABO incompatible | 327 (56.0) | 99 (29.9) |  |
| Maternal blood type |  |  | 0.002 |
| A | 62 (10.6) | 62 (18.7) |  |
| B | 85 (14.6) | 47 (14.2) |  |
| AB | 8 (1.4) | 9 (2.7) |  |
| O | 429 (73.5) | 213 (64.4) |  |
| Neonatal blood type |  |  | < 0.001 |
| A | 253 (43.3) | 102 (30.8) |  |
| B | 213 (36.5) | 110 (33.2) |  |
| AB | 33 (5.7) | 22 (6.6) |  |
| O | 85 (14.6) | 97 (29.3) |  |
| Blood transfusion |  |  | 0.001 |
| No | 551 (94.3) | 328 (99.1) |  |
| Yes | 33 (5.7) | 3 (0.9) |  |
| Other diagnoses refer to premature infants, expiratory dyspnea, and neonatal infection. | | | |
