# Supplementary material for: A Retrospective Study on Neonatal Jaundice: Early Risk Stratification Value of DAT‐FAT Serological Profiles Confirmed by AET
Source: Kaohsiung J Med Sci. 2026 Jun 17:e70253. Online ahead of print. doi: 10.1002/kjm2.70253 (PMC13399740; doi:10.1002/kjm2.70253)
Supplement: Supplementary file 3 — Table S2: Logistic Regression Model Analysis of Jaundice Severity in Subgroups. [file KJM2-9999-e70253-s002.docx]

**Table S2 Logistic Regression Model Analysis of Jaundice Severity in Subgroups**

|  | | Estimate | Std. Error | Wald | Sig. | 95% CI | |
| --- | --- | --- | --- | --- | --- | --- | --- |
|  |  |  |  |  |  | Lower Bound | Upper Bound |
| Threshold | Mild | -3.580 | 0.522 | 47.130 | 0.000 | -4.603 | -2.558 |
|  | Moderate | -1.675 | 0.444 | 14.219 | 0.000 | -2.546 | -0.804 |
| Location | Group 1 | -2.099 | 0.542 | 15.016 | 0.000 | -3.161 | -1.037 |
|  | Group 2 | 2.781 | 0.839 | 10.988 | 0.001 | 1.137 | 4.426 |
|  | Group 3 | 0.031 | 0.491 | 0.004 | 0.950 | -0.931 | 0.993 |
|  | Group 4 | Reference group | - | - | - | - | - |

**Abbreviations:** CI: Confidence Interval. **Note:** Group 1: Direct Antiglobulin Test positive, Free Antibody Test negative, Antibody Elution Test positive; Group 2: Direct Antiglobulin Test positive, Free Antibody Test positive, Antibody Elution Test positive; Group 3: Direct Antiglobulin Test negative, Free Antibody Test positive, Antibody Elution Test positive; Group 4: Direct Antiglobulin Test negative, Free Antibody Test negative, Antibody Elution Test positive.
