# Supplementary material for: A Retrospective Study on Neonatal Jaundice: Early Risk Stratification Value of DAT‐FAT Serological Profiles Confirmed by AET
Source: Kaohsiung J Med Sci. 2026 Jun 17:e70253. Online ahead of print. doi: 10.1002/kjm2.70253 (PMC13399740; doi:10.1002/kjm2.70253)
Supplement: Supplementary file 4 — Table S3: Univariate and Multivariate Logistic Regression Analyses of Prolonged Hospitalization in Neonates with Jaundice. [file KJM2-9999-e70253-s004.docx]

**Table S3 Univariate and Multivariate Logistic Regression Analyses of Prolonged Hospitalization in Neonates with Jaundice**

| **Variable** | **Univariate OR (95% CI)** | **Univariate P-value** | **Multivariate OR (95% CI)** | **Multivariate P-value** |
| --- | --- | --- | --- | --- |
| Group2 | 7.549 (2.806-20.308) | < 0.001 | 2.656 (0.896-7.873) | 0.078 |
| Group3 | 4.784 (1.782-12.846) | 0.002 | 2.415 (0.848-6.878) | 0.099 |
| Group4 | 5.376 (1.777-16.260) | 0.003 | 2.481 (0.766-8.036) | 0.130 |
| IVIG | 1.558 (0.932-2.605) | 0.091 | - | - |
| Birth weight | 1.000 (0.999-1.000) | 0.167 | - | - |
| Mode of delivery | 0.943 (0.614-1.446) | 0.787 | - | - |
| Maternal pregnancy times | 1.035 (0.868-1.235) | 0.701 | - | - |
| Mother-infant blood type | 1.054 (0.715-1.554) | 0.790 | - | - |
| WBC on admission | 1.020 (0.943-1.104) | 0.614 | - | - |
| Hb on admission | 1.009 (0.999-1.020) | 0.084 | - | - |
| DBIL on admission | 1.096 (1.046-1.148) | < 0.001 | 0.989 (0.930-1.051) | 0.711 |
| TBIL on admission | 1.009 (1.006-1.012) | < 0.001 | 1.005 (1.001-1.009) | 0.028 |
| Phototherapy | 4.135 (2.521-6.783) | < 0.001 | 2.197 (1.153-4.187) | 0.017 |
| Blood transfusion | 2.228 (1.033-4.803) | 0.041 | 1.771 (0.778-4.033) | 0.173 |
| Gender | 0.724 (0.495-1.059) | 0.096 | - | - |
| Neonatal blood type | 1.054 (0.715-1.554) | 0.790 | - | - |
| Gestational week | 0.817 (0.701-0.951) | 0.009 | 0.828 (0.703-0.976) | 0.024 |
| Postnatal age on admission | 0.983 (0.903-1.070) | 0.692 | - | - |

Abbreviations: OR: Odds Ratio; CI: Confidence Interval; IVIG: Intravenous Immunoglobulin; WBC: White Blood Cell; Hb: Hemoglobin; DBIL: Direct Bilirubin; TBIL: Total Bilirubin. Note: Group 1: Direct Antiglobulin Test positive, Free Antibody Test negative, Antibody Elution Test positive; Group 2: Direct Antiglobulin Test positive, Free Antibody Test positive, Antibody Elution Test positive; Group 3: Direct Antiglobulin Test negative, Free Antibody Test positive, Antibody Elution Test positive; Group 4: Direct Antiglobulin Test negative, Free Antibody Test negative, Antibody Elution Test positive. Group 1 was used as the reference group.
